# HCMV Activates the IL-6-JAK-STAT3 Axis in HepG2 Cells and Primary Human Hepatocytes

**DOI:** 10.1371/journal.pone.0059591

**Published:** 2013-03-26

**Authors:** Quentin Lepiller, Wasim Abbas, Amit Kumar, Manoj K. Tripathy, Georges Herbein

**Affiliations:** Department of Virology, University of Franche-Comté, EA 4266 “Pathogens & Inflammation”, SFR FED4234, CHU Besançon, Besançon, France; McMaster University, Canada

## Abstract

**Objectives:**

There has been increased interest in the possible role of human cytomegalovirus (HCMV) in carcinogenesis during the last decade. HCMV seroprevalence was enhanced in patients with hepatocellular carcinoma (HCC) but a possible relationship between HCC and HCMV infection remained to be assessed. The aim of this work was to investigate the pro-tumor influence of HCMV on primary human hepatocytes (PHH) and HepG2 cells.

**Methods:**

Following infection of PHH and HepG2 cells by two different strains of HCMV, we measured the production of IL-6 in culture supernatants by ELISA and the protein levels of STAT3, pSTAT3, JAK, cyclin D1, survivin, p53, p21, and Mdm2 by western Blotting in infected and uninfected cells. Cell proliferation and transformation were investigated using Ki67Ag expression measurement and soft-agar colony formation assay respectively.

**Results:**

Infection of HepG2 cells and PHH by HCMV resulted in the production of IL-6 and the subsequent activation of the IL-6R-JAK-STAT3 pathway. HCMV increased the expression of cyclin D1 and survivin. Cell proliferation was enhanced in HepG2 and PHH infected with HCMV, despite a paradoxical overexpression of p53 and p21. More importantly, we observed the formation of colonies in soft agar seeded with PHH infected with HCMV and when we challenged the HepG2 cultures to form tumorspheres, we found that the HCMV-infected cultures formed 2.5-fold more tumorspheres than uninfected cultures.

**Conclusion:**

HCMV activated the IL-6-JAK-STAT3 pathway in PHH and HepG2 cells, favored cellular proliferation, induced PHH transformation and enhanced HepG2 tumorsphere formation. Our observations raise the possibility that HCMV infection might be involved in the genesis of hepatocellular carcinoma.

## Introduction

Viruses can induce chronic inflammation and lead to cellular transformation. For example, the hepatitis B and C viruses (HBV and HCV) trigger hepatocellular carcinoma (HCC), the most common primary liver cancer. In addition to HBV and HCV infections, non-infectious inflammatory states, such as the chronic inflammation induced by alcohol consumption and hereditary iron overload, can also contribute to HCC [1]. IL-6 levels are elevated in the serum of patients with these chronic liver diseases and increase even more in patients who develop HCC [2], [3]. Interestingly, high serum levels of IL-6 helped to predict the development of HCC in both HBV and HCV infected patients [4], [5]. Production of IL-6 is triggered by TNF alpha and IL-1, by bacterial products (LPS), or by viral infections, including human cytomegalovirus (HCMV) [6], [7]. Binding of IL-6 onto the IL-6 receptor (IL-6R) is followed by activation of the Janus kinases (JAKs), which in turn phosphorylates and thus activates the transcription factor “signal transducer and activator of transcription-3” (STAT3) [8]. Phosphorylated STAT3 dimerizes and then localizes to the nucleus, where it induces, among others, the genes encoding cyclin D1, survivin, and Bcl-2, thereby promoting growth and proliferation, and preventing apoptosis [9], [10].

HCMV is an opportunistic, species-specific herpes virus that infects a large proportion of the population worldwide and results in an asymptomatic latent infection in healthy subjects. However, HCMV infection can lead to severe diseases in the absence of an effective immune response, especially in patients with AIDS and in immunocompromised solid-organ and bone marrow allograft recipients [11]. During the last decade, by using highly sensitive techniques, several groups have detected the presence of HCMV in a large proportion of glioma, colon cancers, breast cancers, prostate cancers, skin cancers, salivary gland cancers, and medulloblastomas [12], [13], [14], [15], [16], [17], [18]. Moreover, HCMV could act as an “oncomodulator” both on the tumor cells and the microenvironment to promote inflammation, cell cycle progression, immune escape, tumor invasivity, angiogenesis, and survival [19], [20].

In this study, we report that HCMV induced the release of IL-6 and activated the IL-6R-JAK-STAT3 axis in HCMV-infected HepG2 cells and PHH. Moreover, cyclin D1 and survivin were upregulated in HCMV-infected cells. Despite the overexpression of the tumor suppressor p53, we noticed an enhanced proliferation in HepG2 cells and PHH infected with HCMV. Additionally, we observed the formation of colonies in soft agar seeded with PHH infected with HCMV and enhanced tumorsphere formation in HCMV-infected HepG2 cells, indicating that HCMV infection might be involved in the genesis of hepatocellular carcinoma.

## Materials and Methods

### Reagents

Anti-STAT3, anti-pSTAT3, anti-Mdm2, anti-cyclin D1, anti-Ki-67 PE and anti-IE (pp72) HCMV Ag antibodies were purchased from Santa Cruz Biotechnology (Santa Cruz, CA). The anti-IE-1(pp72) HCMV antibody was directed against the exon 4 of IEpp72 (6E1: sc-69834). Anti-US28 (vC-17: sc-28042), anti-pp65 (1-L-11: sc-52401) and anti-65 kD structural late antigen (0896: sc-58116) antibodies were purchased from Santa Cruz Biotechnology. Isotype control (IgG-PE) was purchased from BD pharmingen (BD Biosciences San Jose, CA, USA). Anti-JAK, anti-p53, anti-p21waf, and anti-survivin were purchased from Cell Signaling Technologies (Beverly, MA). Anti-beta-actin antibody was purchased from Sigma-Aldrich (St. Louis, MO). The STAT3 inhibitor WP1066 and the JAK inhibitor pyridone 6 were purchased from Merck (Darmstadt, Germany). Neutralising anti-EGFR and anti-IL-6R antibodies were purchased from Millipore (Billerica, MA) and R&D Systems (Minneapolis, MN) respectively. Recombinant glycoprotein gB was purchased from Abcam (Cambridge, UK). Ganciclovir was purchased from Roche (Basel, Switzerland).

### Cell culture

HepG2 cells were obtained from the European Collection of Cell Cultures (ECACC, Porton Down, UK) and PHH from Kaly-Cell (Strasbourg, France). HepG2 cells were cultivated in Eagle's Minimum Essential Medium (EMEM) supplemented with 10% fetal bovine serum, 1% non-essential amino acids (Sigma-Aldrich, St. Louis, MO), penicillin (100 IU/ml), and streptomycin (100 microg/ml). PHH were cultivated in serum-free Dulbecco's Modified Eagle Medium supplemented with L-glutamine (2 mmol/l), insulin (4 microg/ml), dexamethasone (1 micromol/l), and gentamycin (50 mg/l). The PHH were free of HCV, HBV, HIV, and HCMV as determined by highly sensitive PCR and RT-PCR assays (Abbott; Argene). Cell viability assay was performed as previously described [21]. IL-6 production was measured in culture supernatants using an ELISA kit (Human IL-6 Quantikine ELISA kit, R&D Systems, Minneapolis, MN). Quantification of HCMV titers in cell culture supernatants was performed by real-time PCR as previously described [11].

### HCMV infection of HepG2 cells and primary human hepatocytes

Cell-free virus stock was prepared by propagating two strains of HCMV, the laboratory strain AD169 and a clinical isolate, HCMV-DB, in MRC5 human fibroblasts as described previously [11], [21]. AD169 is a highly-passaged laboratory strain of HCMV originally isolated from the adenoids of a child [22]. The clinical isolate HCMV-DB was isolated from a cervical swab specimen from a 30-year-old pregnant woman [21]. MRC5 human fibroblasts were cultured in EMEM with 10% FBS, penicillin (100 IU/ml), and streptomycin (100 microg/ml). HepG2 cells and PHH were infected at different multiplicities of infection (MOI) for 2 h at 37°C, washed thoroughly, and covered with fresh medium. Where specified, cells were treated with ganciclovir (5 microg/ml) during infection with HCMV. Ultraviolet (UV)-inactivated HCMV (UV-HCMV; 1200 microJ.cm^−2^, 15 min) was used as control. Supernatants were clarified by centrifugation and stored at −80°C until use. Virus titers were determined by plaque-forming assay in MRC5 human fibroblasts as described previously [23].

### RT-PCR assay

Briefly, total RNA was extracted from HepG2 cells with RNeasy mini kit (Qiagen, Hilden, Germany). RNA (2 microg) was reverse transcribed into cDNA with Superscript III RT (life technologies) using oligo (dT) primers. The RT product (2 microl) was used to perform PCR of IE-1 exon-4 and US28 transcripts with following pairs of primers: IE-1 forward primer - 5′ CTCTGTCCTCAGTAATTGTGGCTG 3′ IE-1 Reverse primer: 5′ GCAACTTCCTCTATCTCAGACACTG3′. US 28 forward primer: 5′ AGCGTGCCGTGTACGTTAC-3′ US28 reverse primer: 5′ - ATAAAGACAAGCACGACC - 3′. The beta-globin gene was amplified as an internal control (sense, 5′-TCCCCTCCTACCCCTACTTTCTA-3′; antisens, 5′-TGCCTGGACTAATCTGCAAGAG-3′). The PCR product was analysed on a 2% agarose gel and visualized after staining with ethidium bromide. For quantitative RT-PCR, 2 microl cDNA product was used in 50 microl cDNA amplification reaction with 300 nM of IE-1 and US-28 primers (mentioned earlier) along with syber green PCR master mix (Qiagen, Hilden, Germany). The reaction were set up in MicroAmp optical 96-well reaction plate (Applied Biosystems), sealed and cycled on Stratagene MX3005P realtime qPCR system (Stratagene) with 95°C for 10 min, followed by 40 cycles at 95°C for 15 sec and annealing/extension on 60°C for 1 min. The DeltaCt values were calculated by subtracting the Ct values of HCMV infected cells from Ct values of uninfected or UV inactivated HCMV infected cells.

### Viral entry assay

Viral entry into HepG2 cells, PHH and MRC5 fibroblasts was assayed as described previously [21]. Cells were incubated at 37°C with HCMV-AD169 at MOIs of 1 and 10 for 2 h and washed three times with PBS. Cells were treated with 0.25% trypsin for 10 min to release the virions that had adhered to the surface but had not entered the cell. The cells were pelleted and washed once with serum neutralization solution and three times with PBS. DNA was extracted from the cell pellet using the KingFisher automatic instrument (Thermo Labsystems) and a QIAamp kit (Qiagen) according to the recommendations of the manufacturers. Samples of eluted DNA were analyzed by PCR using primers specific for the MIEP of HCMV (sense, 5′-TGGGACTTTCCTACTTGG-3′; antisense, 5′-CCAGGCGATCTGACGGTT-3′). The beta-globin PCR gene was used as an internal control (sense, 5′-TCCCCTCCTACCCCTACTTTCTA-3′; antisens, 5′-TGCCTGGACTAATCTGCAAGAG-3′). The amplification products were resolved by 2% agarose gel electrophoresis and visualized by ethidium bromide staining.

### Western blotting

Cellular extracts of HepG2 cells or PHH, either uninfected or infected with HCMV, were used to examine STAT3, pSTAT3, cyclin D1, survivin, JAK, p53, p21waf, Mdm2, HCMV pp72 IE antigen, HCMV US28 antigen, HCMV pp65 antigen, HCMV 65 kD structural late antigen and beta-actin protein expression by Western blotting as described previously [21].

### Cell proliferation

For proliferation assays, HepG2 cells and PHH were left uninfected or were infected with HCMV. Proliferation was measured using the MTT cell proliferation assay kit (Cayman Chemical, Ann Arbor, MI). The Ki67 Ag was measured by intracellular flow cytometry as described previously [24].

### Soft-agar colony formation assay

Soft-agar colony formation by PHH, HepG2 cells and MRC-5 cells uninfected or infected using live or inactivated HCMV (heat-inactivated or UV-inactivated virus), was assayed using Cell Biolabs CytoSelect Cell Transformation Assay kit (Colorimetric assay, CB135; Cell Biolabs Inc., San Diego, CA) and the manufacturer's protocol. Starting 1 day postinfection, cells were incubated for 7 days (HepG2 cells, MRC5) or 2 days (PHH) in the semisolid agar medium. Colonies were observed under an Olympus microscope (magnification ×100 and 200). The 125 microl of 1× Matrix Solubilization Solution was added and thoroughly mixed to each well. 100 microl of the mixture was transferred to a 96-well microtiter plate. Then 10 microl of MTT solution was added to each well and the plate was incubated for 4 h at 37°C and 5% CO_2_. Then 100 microl of detergent solution was added to each well. The plate was incubated in the dark for 4 h at room temperature, with gentle shaking and measure the absorbance at 570 nm in 96-well microtiter plate reader using Multiskan Ex (Thermo Electron Corporation, France).

### Tumorsphere assays

Tumorsphere formation by uninfected HepG2 cells or by HepG2 cells infected using live or UV-inactivated HCMV, was assayed using StemXVivo serum-free tumorsphere media (R&D Systems) supplemented with heparin (2 U/ml) (Sigma) and hydrocortisone (0.5 microg/ml) (Sigma) following the manufacturer's protocol. Starting 1 day postinfection, HepG2 cells were trypsinized with TrypLE™ Express (Life technologies) and resuspended in warmed culture media. The cell suspension was centrifuged at 400× g for 5 min. The liquid was aspirated and the cell pellet was gently resuspended into a single cell suspension with a 5 ml pipette in 2 ml warmed StemXVivo complete culture media. Finally, 10,000 cells were resuspended in 2 ml complete StemXVivo media and transfered to each well of ultra low attachment 6-well plates (Sigma) which were incubated in a 5% CO_2_ incubator at 37°C for 9–10 days. The number of tumorspheres larger than 60 microns was counted.

### Statistical analysis

The reported values are the means and SD or SEMs of independent experiments. Statistical analysis was performed using the student's t test, and differences were considered significant at a value of P<0.05. Microsoft Excel was used to construct the plots.

## Results

### HCMV increases secretion of IL-6 by HepG2 cells and PHH

**Figure 1:**
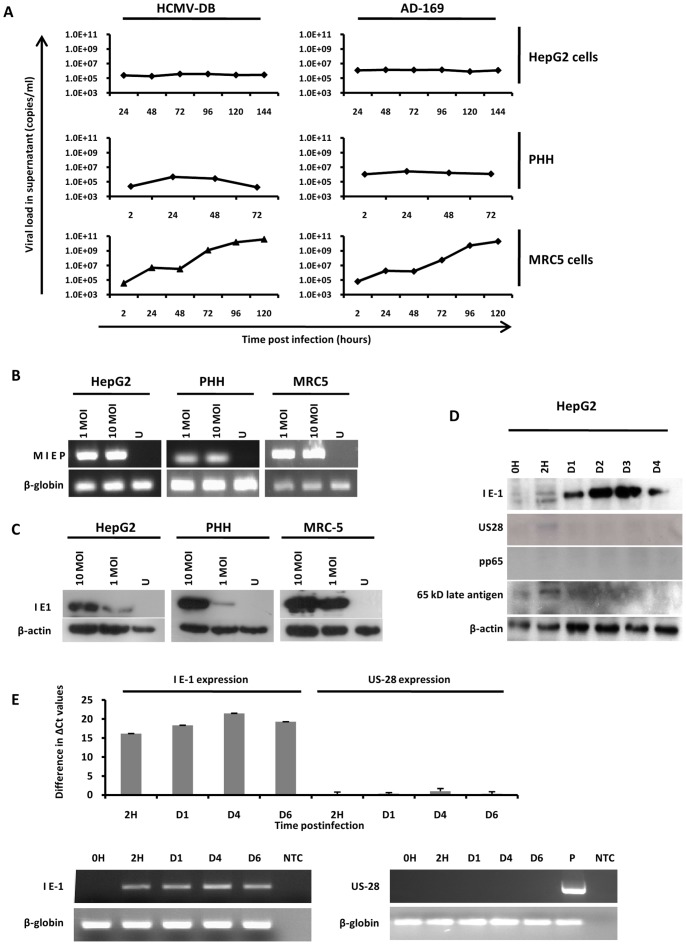
Growth curves of HCMV in HepG2 cells and PHH. (A) *Growth curves of HCMV in HepG2 cells, PHH and MRC5 cells*. HepG2 cells, PHH, and MRC5 cells were left uninfected or infected with HCMV strains AD169 and HCMV-DB (MOI = 1). Inocula were left in place for 2 hours and then removed with three washes of EMEM without serum. Viral titers were determined in the culture supernatants at the indicated times post-infection by real-time PCR. Results are representative of two independent experiments. (B) *Viral entry into HepG2 cells, PHH and MRC5 cells*. Uninfected cells and cultures infected with HCMV-AD169 at the indicated MOI for 2 hours were treated with trypsin for 10 min and then washed. Samples of extracted DNA were analyzed by PCR using primers specific for the MIEP of HCMV and for beta-globin (internal loading control). The amplification products were resolved by 2% agarose gel electrophoresis and visualized by ethidium bromide staining. Results are representative of two independent experiments. (C) *Detection of IE1 pp72 HCMV antigen in infected HepG2 cells and PHH*. HepG2 cells (6×10^6^ cells) and PHH (2×10^6^ cells) were left uninfected or infected with HCMV-AD169 (MOI = 1 and 10). IE1 pp72 HCMV antigen expression was measured at day 3 post-infection by Western blotting as described in the Materials and Methods section. beta-actin was used as control. Results are representative of two independent experiments. (D) *Detection of IE1 pp72, but not US28, pp65 antigen and 65-kD structural late antigen in infected HepG2 cells*. HepG2 cells (6×10^6^ cells) were left uninfected or infected with HCMV-AD169 (MOI = 1). IE1 pp72, US28, pp65 and 65-kD structural late HCMV antigen expression was measured up to day 4 post-infection by Western blotting as described in the Materials and Methods section. beta-actin was used as control. Results are representative of two independent experiments. (E) *Detection of IE1 pp72 transcript, but not of US28 transcript, in HCMV-infected HepG2 cells*. HepG2 cells (6×10^6^ cells) were left uninfected or infected with HCMV-AD169 (MOI = 1). IE1 pp72 and US28 transcript expression was measured up to day 6 post-infection by RT-PCR assay as described in the Materials and Methods section. beta-globin was used as control. Results represent means (± SD) of two independent experiments. IE: Immediate Early; MIEP: Major immediate-early promoter; MOI: Multiplicity of infection; PHH: Primary human hepatocytes; U: Uninfected; P: positive control (extract of MRC5 cells infected with HCMV-AD169).

We infected HepG2 cells and PHH with HCMV strains AD169 and HCMV-DB. We did not observe a highly productive infection of HCMV in these two cell types (Fig. 1A), indicating restricted and/or limited replication of HCMV. By contrast both HCMV strains replicated efficiently in MRC5 fibroblasts (Fig. 1A). To assess the possibility that blocked viral entry influenced the differences in the viral titers, viral entry was assayed in HepG2 cells, PHH and MRC5 fibroblasts through the detection of the intracellular HCMV major immediate early promoter (MIEP). As shown in Fig. 1B, viral entry was similar in all three cell types, indicating efficient entry of HCMV into HepG2 cells and PHH. Using western blotting, the expression of the immediate early 1 (IE1) HCMV phosphoprotein pp72 was observed in infected HepG2 cells and PHH, but not in uninfected cells (Fig. 1C). We then assessed the detection of the immediate early protein IE1 pp72, the early protein US28 and the late proteins pp65 and 65 kD structural late antigen in HCMV-infected HepG2 cells using western blotting. We detected only the immediate early viral protein IE1, but neither the subsequently expressed US28 protein nor any of the late viral proteins (Fig. 1D). Our data indicate that most probably only part of the HCMV viral cycle occurs in infected HepG2 cells, and that HCMV infection does not proceed beyond IE expression in these cells. In agreement with the detection of IE1 pp72 protein, we detected IE1 transcripts in cellular extracts of HCMV-infected HepG2 cells (Fig. 1E). By contrast, neither US28 protein nor US28 transcript were detected following infection of HepG2 cells with HCMV (Figs. 1D and 1E).

**Figure 2:**
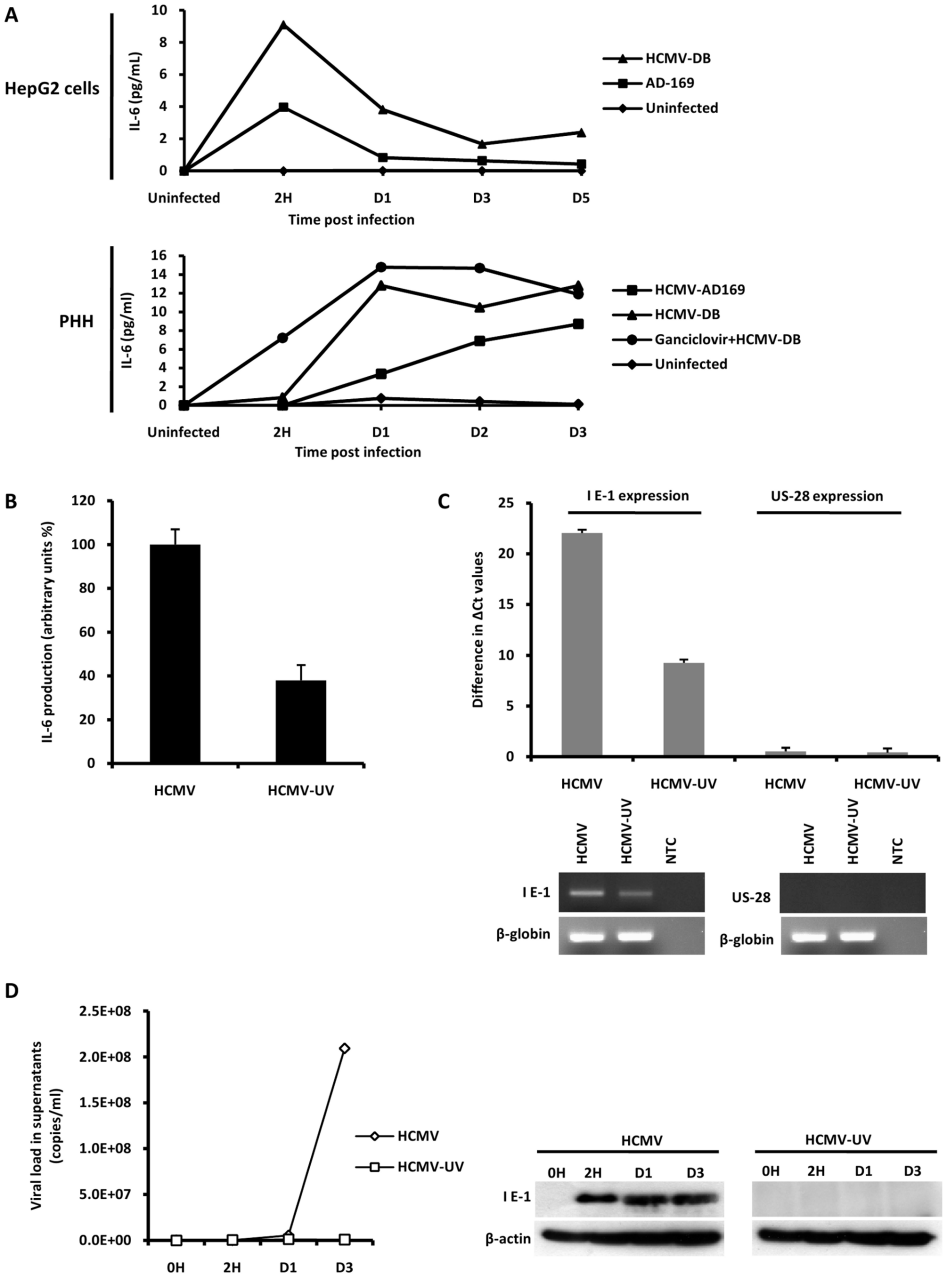
HCMV induces secretion of IL-6 by HepG2 cells and PHH. (A) *ELISA quantification of IL-6 levels in the culture supernatants of HepG2 cells and PHH left uninfected or infected with HCMV strains AD169 and HCMV-DB* (MOI = 1). Where specified, cells were treated with ganciclovir (5 microg/ml). Results are representative of two independent experiments. (B) *Decreased IL-6 production in culture supernatants of HepG2 cells treated with UV-inactivated HCMV in comparison cells infected with live HCMV*. HCMV strain AD169 was used at a MOI = 1 and IL-6 production measured at day 3 post-infection was expressed arbitrarily as 100% in cell cultures infected with live virus. Results represent means (± SD) of two independent experiments. (C) *Decreased IE1 transcript expression in HepG2 cells treated with UV-inactivated HCMV in comparison with live HCMV*. HCMV strain AD169 was used at a MOI = 1 and IE1 and US28 transcripts were amplified by RT-PCR. Difference in DeltaCt values of two independent experiments is shown. Results represent means (± SD) of two independent experiments. (D) *Decreased HCMV replication and IE1 protein expression in MRC-5 cells infected with UV-inactivated HCMV in comparison with cells infected with live HCMV*. HCMV strain AD169 was used at a MOI = 1. Viral titers were determined in the culture supernatants at the indicated times post-infection by real-time PCR. IE1 pp72 antigen expression was measured up to day 3 postinfection by Western blotting, as described in the Materials and Methods section. beta-actin was used as control. Results are representative of two independent experiments.

Because HCMV-infected cells have been reported to produce IL-6 [25], we assessed the secretion of IL-6 by HepG2 cells and PHH infected with HCMV. We observed increased IL-6 production in the supernatants of HepG2 cells and PHH starting as early as 2 h post-infection, with both the HCMV-AD169 and HCMV-DB strains triggering the release of IL-6 (Fig. 2A). The kinetic of IL-6 production was different in HCMV-infected HepG2 cells and PHH (Fig. 2A). Ganciclovir treatment of the cells did not prevent IL-6 production by HCMV (Fig. 2A), indicating that complete viral replication cycle was not required for IL-6 production. In fact, the HCMV stocks used to inoculate the HepG2 cell and PHH cultures were confirmed by ELISA to contain IL-6 at detectable levels (4.6 pg/ml), presumably since HCMV infected MRC5 cells have previously been shown to produce IL-6 [25]. IL-6 production depends on the expression of IE HCMV proteins [26] and the synthesis of HCMV IE proteins is essentially eliminated by UV irradiation of virus stock [27]. Therefore, we analyzed levels of IL-6 following stimulation with live HCMV and UV-inactivated HCMV (UV-HCMV; 1200 microJ.cm^−2^, 15 min) to confirm virus (IE protein)-specificity of IL-6 induction, rather than detection of IL-6 added with the virus inoculum. In comparison with levels observed with live HCMV, 62% decrease in IL-6 production was observed following stimulation with UV-HCMV (Fig. 2B). In agreement with the 62% decrease of IL-6 production in HepG2 cells infected with UV-HCMV, we observed a 58% decrease of IE1 transcript in these cells (Fig. 2C), suggesting a link between IE1 gene expression and IL-6 production in HepG2 cells. We did not detect significant US28 transcripts in HepG2 cells infected with live and UV-inactivated HCMV (Fig. 2C). To assess the extent of HCMV inactivation by UV treatment, we infected MRC-5 with UV-treated virus. We observed that UV-treatment almost completely abolished virus infectivity and IE1 expression (Fig. 2D). Taken together, these data suggest that the induction of IL-6 was at least in part dependent on viral replication cycle (probably expression of IE HCMV proteins) in HCMV-infected HepG2 cells and PHH.

### HCMV induces IL-6-mediated JAK-STAT3 activation in HepG2 cells and PHH

**Figure 3:**
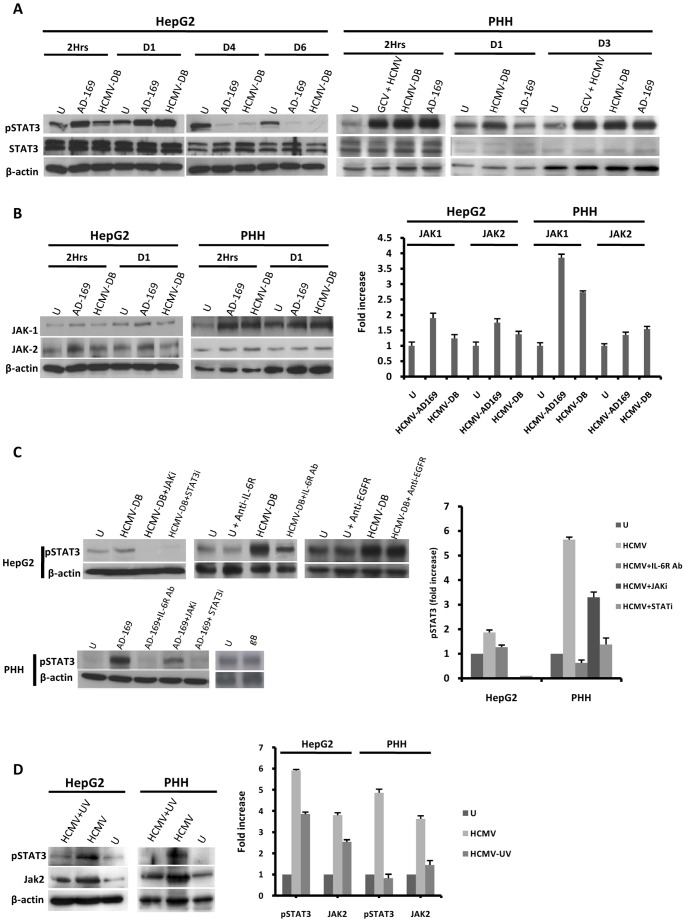
HCMV induces IL-6-mediated activation of the JAK-STAT3 axis in HepG2 cells and PHH. (A) *Time course of STAT3 activation in HepG2 cells and PHH infected with HCMV.* HepG2 cells (6×10^6^ cells) were left uninfected or infected with HCMV strains AD169 and HCMV-DB (MOI = 0.5). PHH (2×10^6^ cells) were left uninfected or infected with HCMV strains AD169 and HCMV-DB (MOI = 1). STAT3 activation was measured by Western blotting as described in the Materials and Methods section. Unphosphorylated STAT3 and beta-actin were used as controls, and ganciclovir was used at a concentration of 5 microg/ml. (B) *Time course of JAK1/JAK2 activation in HepG2 cells and PHH infected with HCMV.* HepG2 cells (6×10^6^ cells) were left uninfected or infected with HCMV strains AD169 and HCMV-DB (MOI = 0.5). PHH (2×10^6^ cells) were left uninfected or infected with HCMV strains AD169 and HCMV-DB (MOI = 1). JAK1/JAK2 activation was measured by Western blotting, and beta-actin was used as an internal control. The histogram shows JAK activation at 2 hours post-infection as quantified using Image J 1.40 software. (C) *STAT3 activation is mediated by the IL-6-JAK pathway in HepG2 cells and PHH infected with HCMV.* HepG2 cells (6×10^6^ cells) and PHH (2×10^6^ cells) were left uninfected or infected with HCMV (MOI = 0.5) in the presence or absence of a JAK inhibitor (1 micromol/l), a STAT3 inhibitor (10 micromol/l), a neutralizing anti-IL-6R mAb (10 microg/ml), and a neutralizing anti-EGFR mAb (20 microg/ml). Cells were left uninfected or incubated with the recombinant HCMV glycoprotein gB (10 microg/ml) for 2 hours. STAT3 activation was measured by Western blotting at day 1 post-infection in PHH incubated with JAK inhibitor, STAT3 inhibitor, anti-IL-6R mAb, and in HepG2 cells incubated with JAK and STAT3 inhibitors. STAT3 activation was measured at 2 hours post-infection in HepG2 cells incubated with anti-IL-6R mAb and anti-EGFR mAb. beta-actin was used as an internal control. The histogram shows STAT3 activation as quantified using Image J 1.40 software. (D) *STAT3 activation is mediated primarily by HCMV in HepG2 cells and PHH*. HepG2 cells (6×10^6^ cells) and PHH (2×10^6^ cells) were left uninfected or infected with HCMV or UV-inactivated HCMV (AD169, MOI = 1). The activation of STAT3 and JAK2 was measured by western blot at day 3 post-infection. beta-actin was used as a control for equal loading. The histogram shows STAT3 and JAK2 activation as quantified using Image J 1.40 software. Results of western-blots are representative of two independent experiments; histograms represent means (± SD) of two independent experiments. Ab: Antibody; EGFR: Epidermal growth factor receptor; GCV: ganciclovir.

IL-6 binds to the IL-6 receptor (IL-6R) to activate STAT3 signaling [6]. Therefore we assessed the phosphorylation status of STAT3 in HepG2 cells and PHH infected with HCMV. Consistent with the presence of IL-6 in the supernatant, STAT3 phosphorylation was markedly increased in HepG2 cells and PHH infected with HCMV compared to mock-infected cells (Fig. 3A). In HepG2 cells, STAT3 phosphorylation was detected as early as 2 h post-infection, peaked 1 day post-infection, and decreased thereafter (Fig. 3A). In contrast, STAT3 phosphorylation was detected as early as 2 h post-infection in PHH and peaked again at day 3 post-infection (Fig. 3A). Both HCMV-AD169 and HCMV-DB strains activated STAT3 in HepG2 cells and PHH (Fig. 3A). In contrast to infection with UV-HCMV, ganciclovir pretreatment of the cells did not prevent STAT3 activation in PHH infected with HCMV (Fig. 3A and 3D), indicating that STAT3 activation, like IL-6 production, did require early steps of viral replication.

Since cytokine activation of STAT3 is mediated by upstream Janus kinases (JAKs) [6], we assessed the expression of JAK-1 and JAK-2 in HepG2 cells and PHH infected with HCMV. JAK-1 and/or JAK-2 activation was increased in HepG2 cells and PHH infected with AD169 or HCMV-DB compared to mock-infected cells (Fig. 3B). Pretreatment of HCMV-infected HepG2 cells and PHH with a pan-JAK inhibitor and a STAT3 inhibitor greatly reduced STAT3 phosphorylation (Fig. 3C), indicating activation of a JAK-STAT3 axis in HepG2 cells and PHH infected with HCMV. Since the binding of IL-6 to IL-6R activates STAT3, we directly assessed the role of IL-6R in STAT3 activation in HepG2 cells and PHH. HCMV infection induced STAT3 activation in both cell types, whereas incubation of HCMV-infected cells with an IL-6R neutralizing antibody decreased STAT3 phosphorylation (Fig. 3C). In contrast, incubation with an EGF receptor (EGFR) neutralizing antibody did not inhibit STAT3 activation by HCMV in HepG2 cells (Fig. 3C). Moreover, incubation of cells with the recombinant glycoprotein gB, which was previously shown to bind to and activate EGFR-mediated pathways [28], failed to activate STAT3 (Fig. 3C). In contrast to infection with live HCMV, decreased activation of STAT3 and JAK2 was observed in cells treated with UV-inactivated HCMV (Fig. 3D). Our results indicate that in HepG2 cells and in PHH, HCMV-induced STAT3 activation was mediated by autocrine and/or paracrine IL-6 production.

### HCMV increases expression of cyclin D1 and survivin in HepG2 cells and PHH

**Figure 4:**
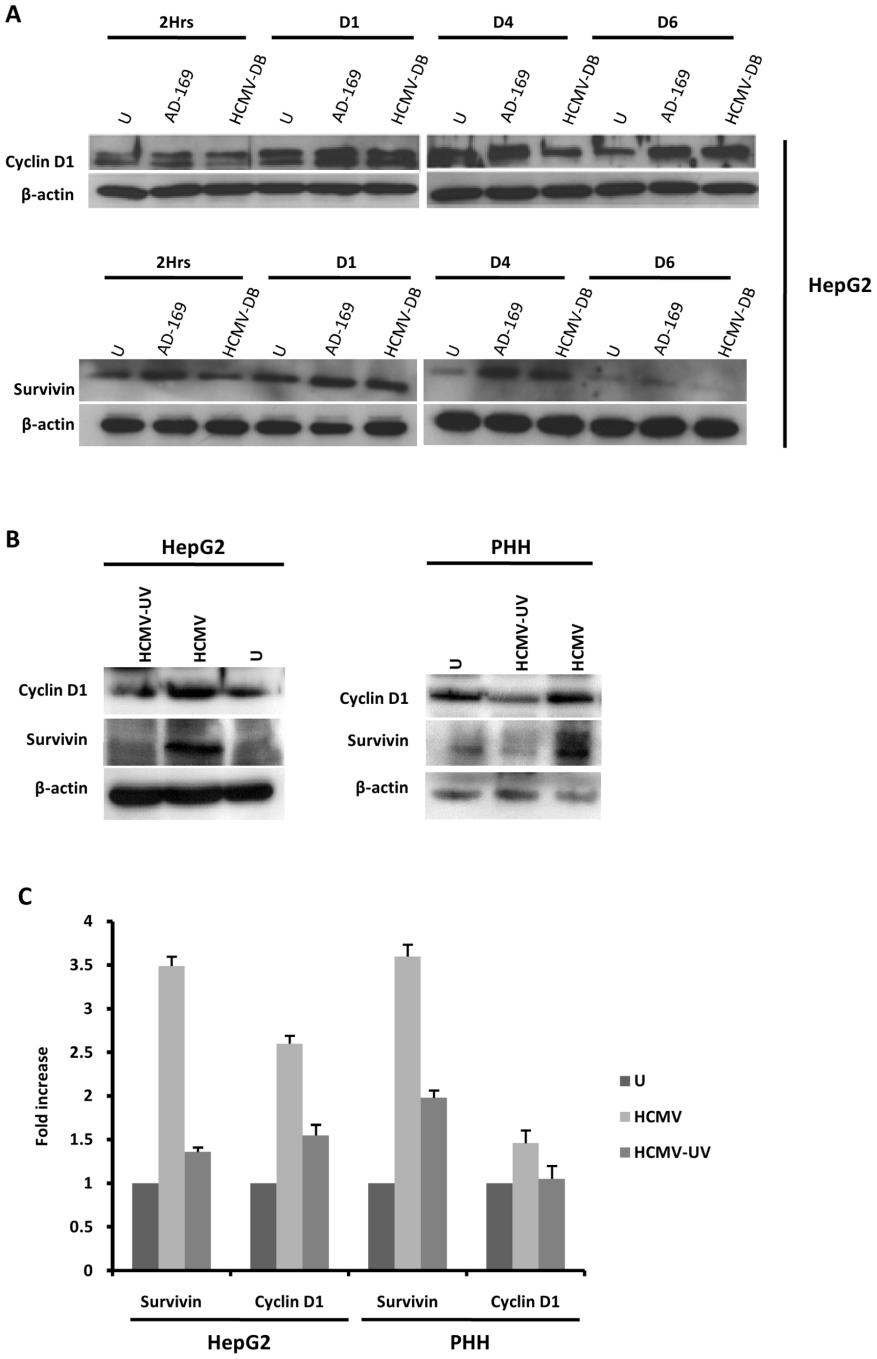
Up-regulation of cyclin D1 and survivin in HepG2 cells and PHH infected with HCMV. (A) *Time course of the expression of cyclin-D1 and survivin in HepG2 cells infected with HCMV.* HepG2 cells (6×10^6^ cells) were left uninfected or infected with HCMV strains AD169 (MOI = 0.5) and HCMV-DB (MOI = 1.0). Cyclin D1 and survivin expression was measured by Western blotting as described in the Materials and Methods, and beta-actin was used as an internal control. (B) *Expression of cyclin-D1 and survivin in PHH and HepG2 cells infected with live HCMV or UV-inactivated HCMV*. HepG2 cells (6×10^6^ cells) and PHH (2×10^6^ cells) were left uninfected or infected with HCMV or UV-inactivated HCMV (AD169, MOI = 0.5). Cyclin D1 and survivin expression was measured by Western blotting as described in the Materials and Methods, and beta-actin was used as an internal control. (C) *Expression of cyclin D1 and survivin is mediated primarily by HCMV in HepG2 cells and PHH.* The histogram shows survivin and cyclin D1 expression at day 3 post-infection as quantified using Image J 1.40 software. Results of western-blots are representative of two independent experiments; histogram represents means (± SD) of two independent experiments.

Cyclin D1 expression is induced during liver regeneration as well as in HCC [29], [30]. Since cyclin D1 overexpression in HCC was mediated by the IL-6-STAT3 axis [31], we assessed the expression of cyclin D1 in HCMV-infected HepG2 cells. We found that HCMV infection enhanced the expression of cyclin D1 in HepG2 cells (Fig. 4A). The up-regulation of cyclin D1 expression was observed with HCMV strains AD169 and HCMV-DB after one day post-infection and was sustained up to 6 days post-infection (Fig. 4A). Since phospho-STAT3 was reported to bind to the promoter of the survivin gene [32], we assessed survivin expression in HCMV-infected HepG2 cells. Survivin expression was upregulated in HepG2 cells infected with HCMV compared to mock-infected control cells (Fig. 4A). Similar results were observed in HCMV-infected PHH (Fig. 4B). Furthermore, cyclin D1 and survivin were expressed at lower levels in HepG2 cells and PHH infected with UV-inactivated HCMV as compared to cells infected with live HCMV (Figs. 4B and 4C).

### HCMV-induced STAT3 activation favors the proliferation of HepG2 cells and PHH

**Figure 5:**
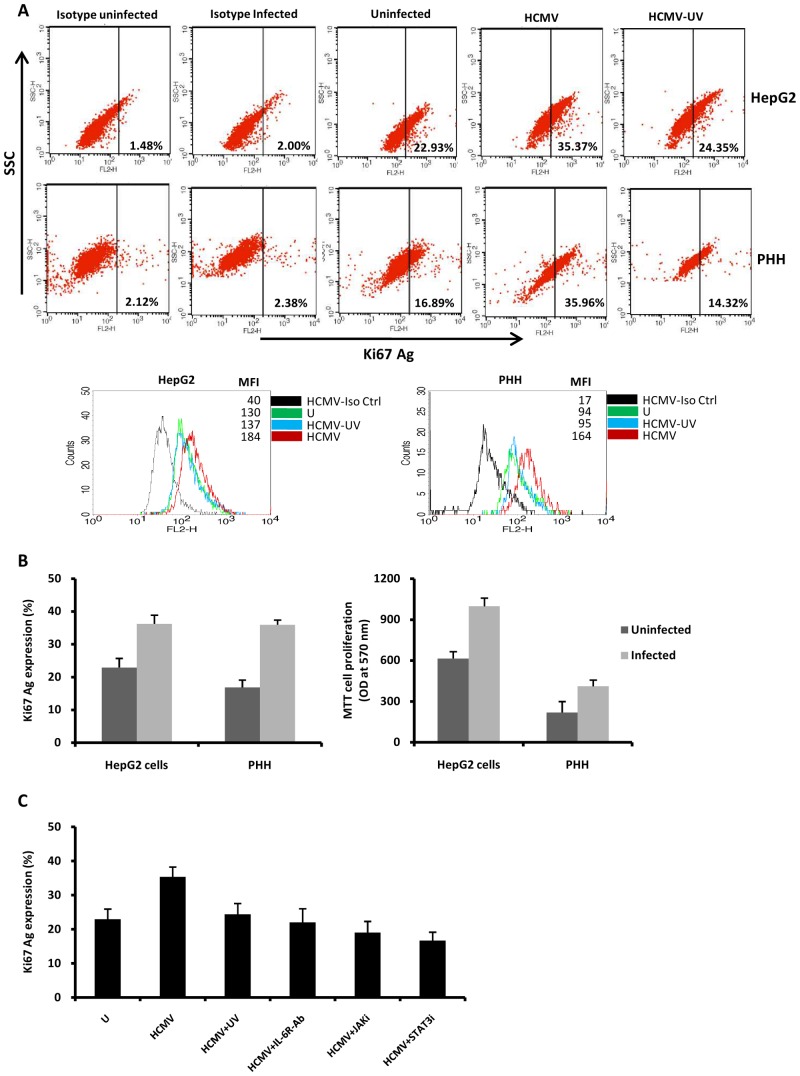
HCMV triggers cell proliferation via IL-6-STAT3 activation in HepG2 cells and PHH. HepG2 cells (6×10^6^ cells) and PHH (2×10^6^ cells) were left uninfected or infected with AD169 (MOI = 0.5) and UV-inactivated HCMV. Cell proliferation was measured by monitoring the expression of Ki-67 Ag using flow cytometry and MTT assay as described in the Materials and Methods. (A) Ki67 Ag expression as measured by flow cytometry. MFI, mean fluorescence intensity. Results are representative of two independent experiments. (B) Histograms show Ki-67 Ag and MTT data from two independent experiments. (C) Increased cell proliferation in HepG2 cells infected with AD169 is blocked by a neutralizing anti-IL-6R mAb (10 microg/ml), a Jak inhibitor (1 micromol/l) and a STAT3 inhibitor (10 micromol/l), and decreased cell proliferation was observed in HepG2 cells infected with UV-inactivated HCMV. Mean values ± SD are representative of two independent experiments.

Since cyclin D1 is involved in cell proliferation, we assessed the proliferation of HepG2 cells and PHH infected with HCMV or UV-inactivated HCMV. We measured the expression of the nuclear antigen Ki67, a hallmark of cell proliferation, by flow cytometric analysis. We observed that HCMV triggered the proliferation of both HepG2 cells and PHH (Fig. 5A,B). The proliferation of HepG2 cells and PHH after HCMV infection was also measured using the MTT assay (Fig. 5B). Pretreatment of HCMV-infected HepG2 cells with a neutralizing anti-IL-6R antibody, a JAK inhibitor, and a STAT3 inhibitor or UV-inactivated HCMV blocked cell proliferation (Fig. 5C), indicating the involvement of the IL-6-JAK-STAT3 axis in the proliferation of HCMV-infected cells.

### HCMV increases expression of p53 and p21 in HepG2 cells

**Figure 6:**
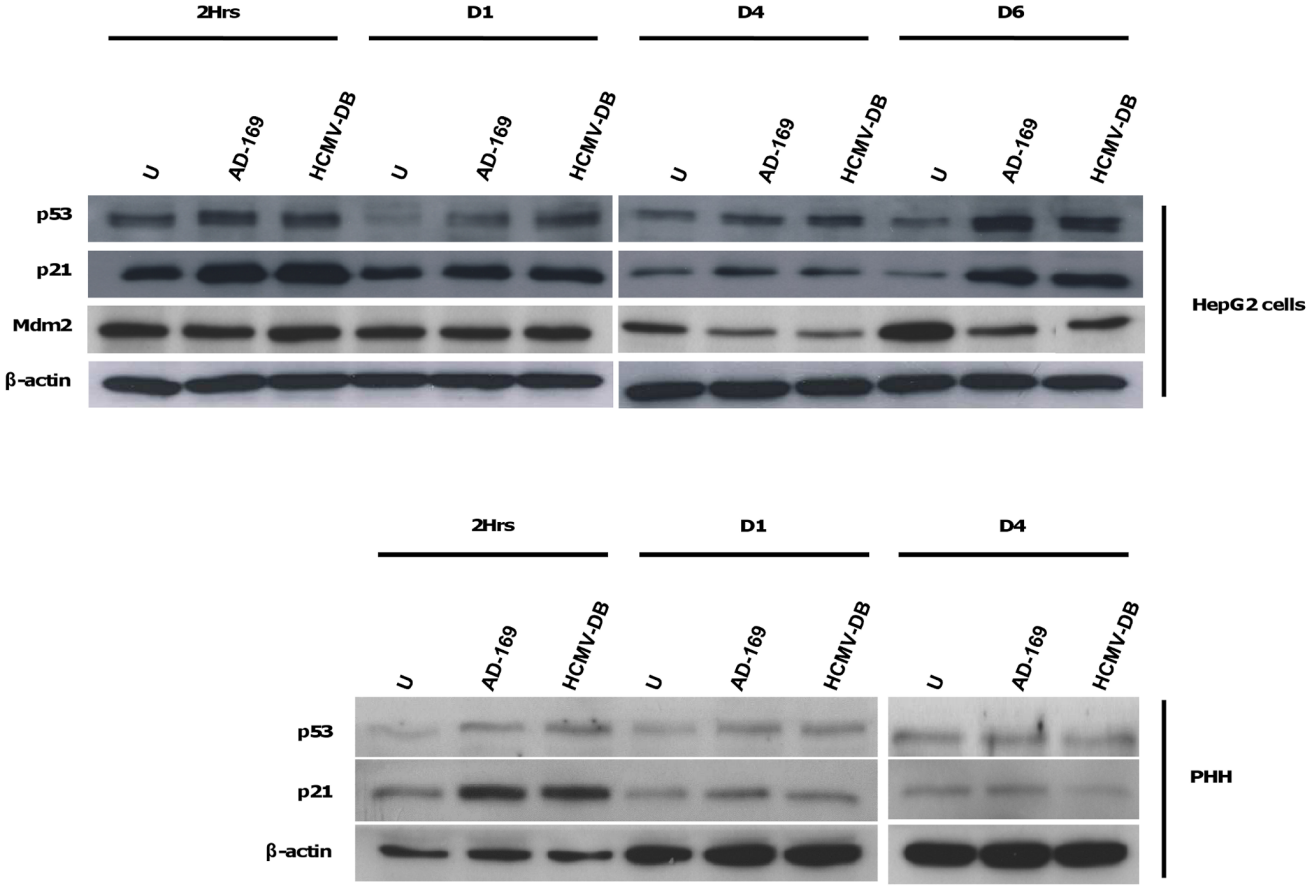
HCMV upregulates p53 and p21 in HepG2 cells and PHH. Time course of p53, p21 and Mdm2 in HepG2 cells and PHH infected with HCMV. HepG2 cells (6×10^6^ cells) and PHH (2×10^6^ cells) were left uninfected or infected with HCMV strains AD169 and HCMV-DB at MOI = 0.5 and 1, respectively. P53, p21 and Mdm2 protein expressions were measured by Western blotting, and beta-actin was used as an internal control. Results are representative of two independent experiments.

In stressed cells, p53 acts as an antitumor protein to induce cell cycle arrest and apoptosis. However, alterations of p53 expression or functions are regularly observed in cancers [33]. Since HCMV increased expression of cyclin D1 and induced the proliferation of HepG2 cells and PHH, we assessed the counterbalanced expression of p53 in these cells. In parallel, we estimated the expressions of the p53-inhibitor Mdm2, and the p53-effector p21 in HCMV-infected HepG2 cells. We observed that both p53 and p21 were overexpressed in HepG2 cells infected with AD169 and HCMV-DB (Fig. 6). The up-regulations of p53 and p21 were noticed as early as 2 hours after infection but predominated at 6 days post-infection. By contrast, Mdm2 expression was downregulated in HCMV-infected HepG2 cells at day 4 and day 6 post-infection (Fig. 6). Enhanced p21 expression was observed at 2 hours post-infection in HCMV-infected PHH (Fig. 6). These results indicate that a p53 apparently adapted response was triggered in HepG2 cells stressed by HCMV infection. However, p53 activation failed to efficiently protect HCMV-infected cells against cell cycle promotion and cellular proliferation.

### PHH infected with HCMV form colonies in soft agar

**Figure 7:**
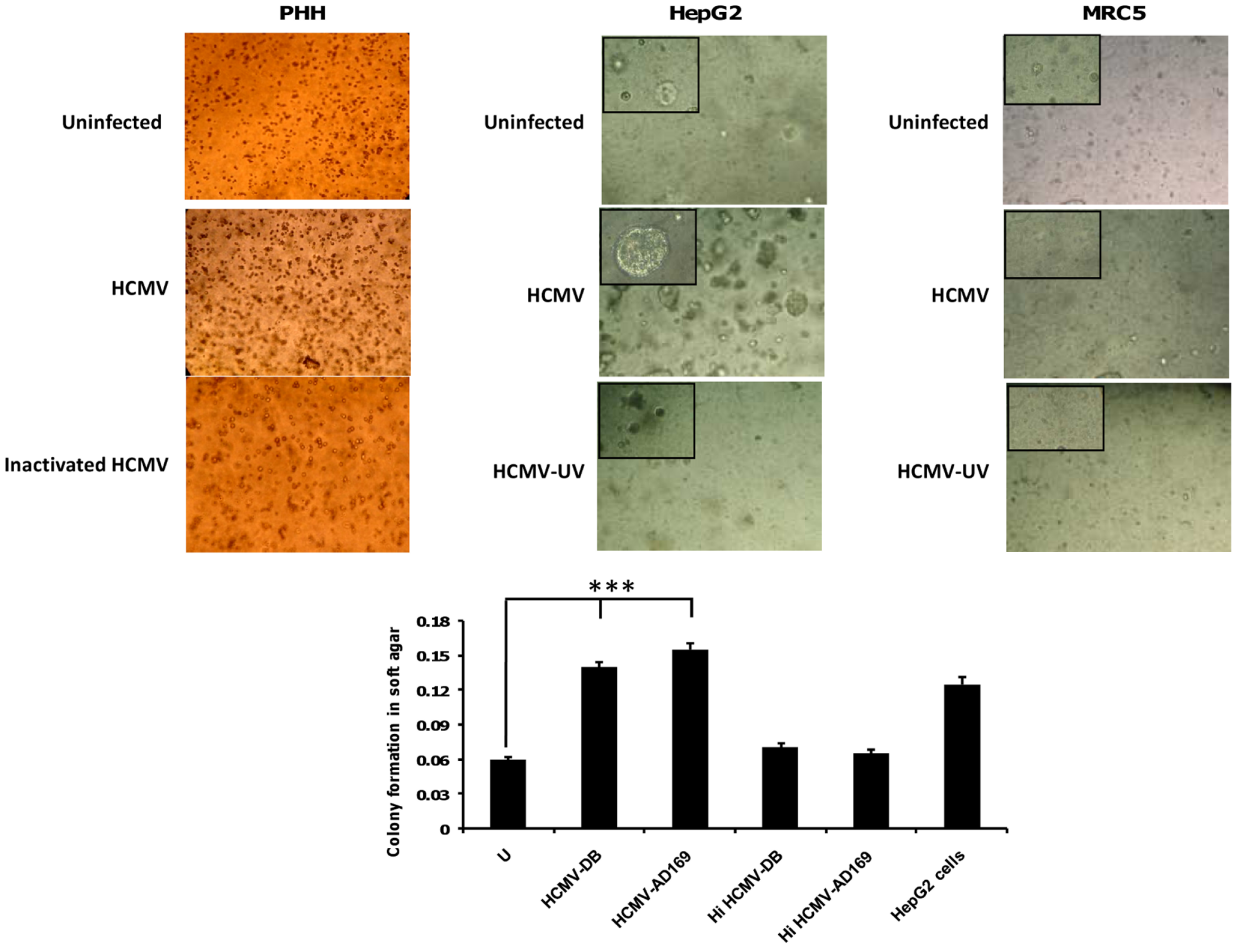
Detection of colony formation in soft agar seeded with HCMV-infected PHH and HepG2 cells. *Upper panel:* Colony formation in soft agar seeded with PHH infected with HCMV-DB, but not with PHH uninfected or infected with heat-inactivated (HI) HCMV. Enhanced formation of colonies in soft agar that had been seeded with HepG2 cells infected with HCMV. None colony formation in soft agar that had been seeded with MRC-5 cells infected with HCMV, UV-inactivated HCMV or uninfected MRC-5 cells. Magnification: 100× (200× in upper left corner of picture). *Lower panel:* The histogram shows quantification of colony formation in soft agar as specified by the manufacturer (Cell Biolabs). Results represent means (± SD) of two independent experiments. ***P<0.001.

Although we detected increased proliferation in PHH following exposure to HCMV, this observation does not indicate definitively that the infected PHH were transformed. We thus used a soft agar assay for colony formation, which is the most stringent assay for detecting the malignant transformation of cells, to directly test whether PHH were transformed following HCMV exposure. On day 1 post-infection with HCMV strains AD169 and HCMV-DB, PHH were cultured in soft agar medium for 2 days. In parallel, uninfected cells and cells infected with heat-inactivated HCMV were cultured as negative controls, and HepG2 cells were cultured as a positive control. After 2 days of culture (i.e. on post-infection day 3), we observed the formation of colonies in soft agar that had been seeded with PHH infected with the HCMV strains HCMV-DB and AD169 (Fig. 7). We also observed enhanced formation of colonies in soft agar that had been seeded with HepG2 cells infected with HCMV (Fig. 7). None colony formation was observed in soft agar that had been seeded with MRC-5 cells infected with HCMV or not (Fig. 7). These results indicate that *in vitro* cellular transformation associated with loss of contact inhibition and anchorage independence occurred in PHH infected with HCMV-DB and AD169.

### Enhanced tumorsphere formation by HCMV-infected HepG2 cells

**Figure 8:**
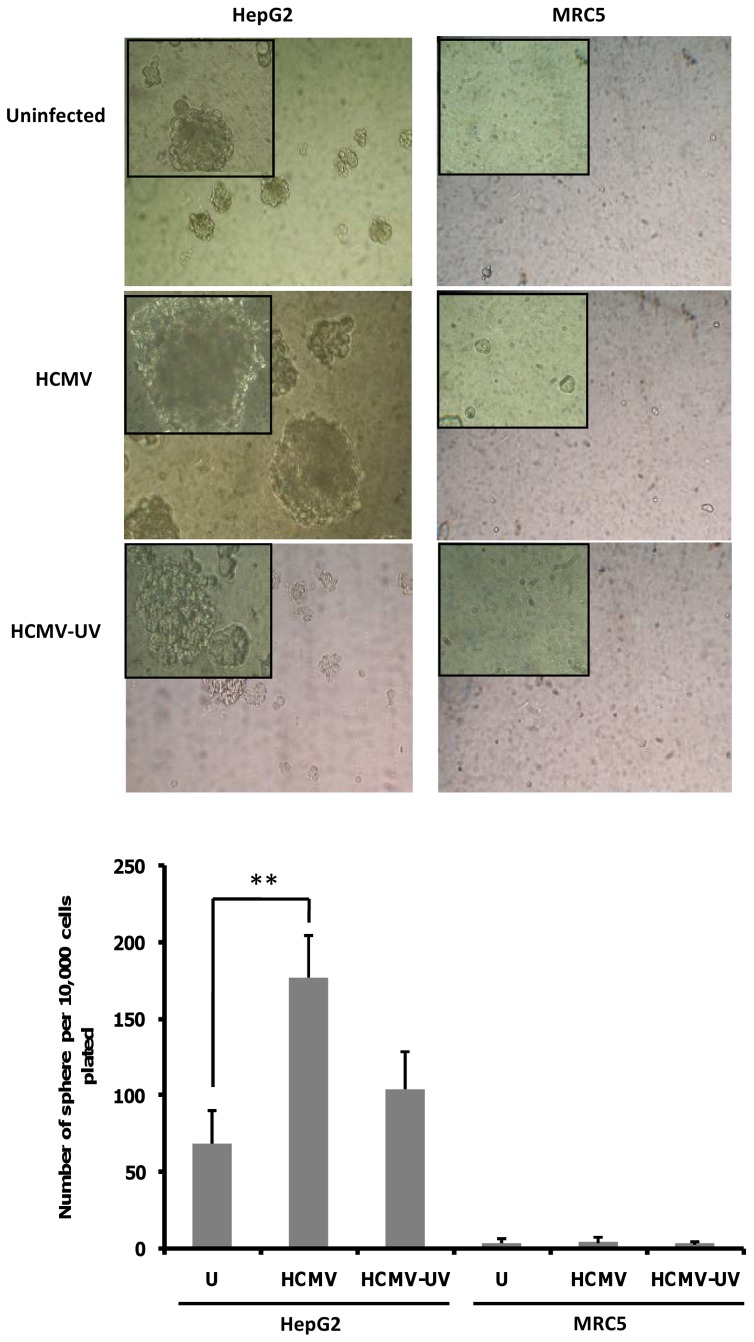
HCMV infection increases HepG2 tumorsphere formation. *Upper panel:* HepG2 cells and MRC-5 cells were infected with HCMV, UV-inactivated HCMV or left uninfected. The tumorsphere formation was assessed at day 9–10 post-infection. Representative phase contrast micrographs of HepG2 tumorspheres are shown. As a negative control, HCMV-infected MRC5 cells did not form tumorspheres. Magnification: 100× (200× in upper left corner of each picture). *Lower panel:* When we challenged the HepG2 cultures to form tumorspheres, we found that HCMV infection formed 2.5-fold more tumorspheres than uninfected cultures. The histogram presents tumorsphere formation presented as the average number of spheres per 10,000 cells plated (± SEM). Results represent three biological replicates. **P<0.01.

Since activation of IL-6/STAT3 axis signaling in cancer stem cells (CSC) enhances proliferation and survival as well as tumor growth in mice, we decided to detect the presence of CSC in HepG2 cells uninfected and infected with HCMV using a tumorsphere formation assay [34], [35]. To determine whether HCMV infection could indeed induce CSC expansion, we infected HepG2 cells with HCMV for 9–10 days and evaluated the proportion of stem-like cells by sphere formation assay. When we challenged these HepG2 cultures to form tumorspheres, we found that HCMV infection formed 2.5-fold more tumorspheres than uninfected cultures (Fig. 8). As a negative control, HCMV-infected MRC5 cells did not form tumorspheres (Fig. 8).

## Discussion

**Figure 9:**
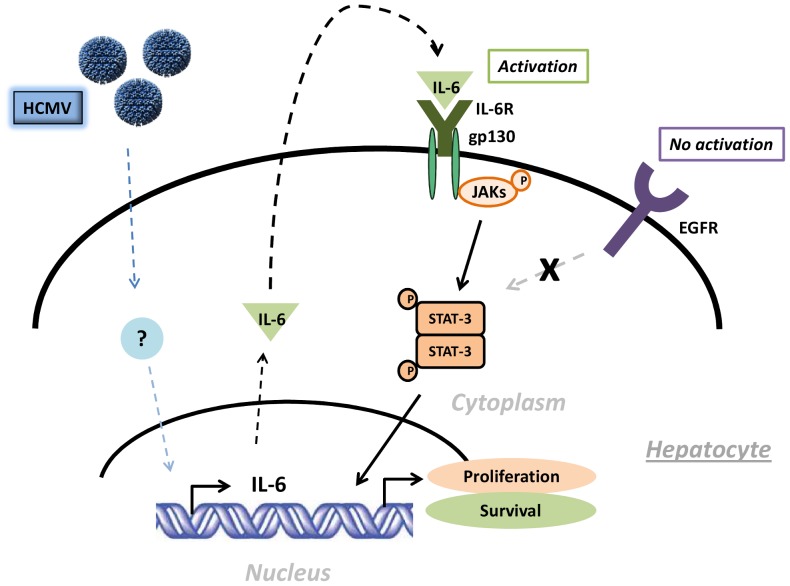
Potential oncogenic effect of the IL-6-JAK-STAT3 axis in PHH.

In this study, we first observed that infection of HepG2 cells and PHH with HCMV resulted in low-level productive viral growth. Further experiments showed that HCMV triggered the activation of the IL-6-JAK-STAT3 axis in HepG2 cells and PHH (Fig. 9). We observed the upregulation of cyclin D1 and survivin, two proteins that contain a STAT3-binding domain in their promoters, in HCMV-infected HepG2 cells and PHH. We also found that HCMV triggers cell proliferation in HepG2 cells and PHH through STAT3 activation. In HCMV-infected HepG2 cells and PHH, the activations of p53 and p21 failed to efficiently counterbalance the proliferative effect of the virus. Finally, we observed the formation of colonies in soft agar seeded with PHH infected with the HCMV strains HCMV-DB and AD169. Taken together, these results indicated that HCMV enhances HepG2 cell and PHH proliferation via the IL-6-JAK-STAT3 pathway, potentially contributing to the development of HCC.

The importance of IL-6 and STAT3 signaling in oncogenesis [9], [10] prompted us to investigate the role of the IL-6-STAT3 axis in HCMV-mediated proliferative signaling. The increase in IL-6 secretion by HCMV-infected HepG2 cells and PHH was associated with increased activation of STAT3 through the upstream activation of JAK. This increase was observed in infected cells, but not in uninfected cells. Using IL-6R-neutralizing antibodies, we showed that HCMV activates the IL-6-JAK-STAT3 signaling axis in an autocrine and/or paracrine manner in both HepG2 cells and PHH. Treatment of cells with STAT3 or JAK inhibitors diminished Ki-67 Ag nuclear labelling, further demonstrating the relevance of the JAK-STAT3 pathway to the HCMV-induced proliferative phenotype. In agreement with our findings, STAT3 is a transcriptional regulator that shows increased activity in solid tumors such as HCC and breast cancers, among others [10], [36]. Recent studies have shown that constitutively active gp130 mutants are responsible for increased STAT3 phosphorylation in HCC [37], and initial reports have demonstrated that inhibition of aberrantly activated STAT3 exerts an antitumor effect in HCC [38]. In addition to JAK-1 [9], IL-6/JAK-2/STAT3 activation and tumor progression in hepatocellular carcinoma has recently been reported [39].

Activation of the IL-6/STAT3 signaling axis depends on the expression of HCMV proteins such as US28 and IE1 [26], [40], [41]. The transient induction of pSTAT3 observed in HCMV infected cells may be dependent on IE1 or US28 proteins expressed by incoming virus. The most likely viral candidate to explain the STAT3 activation in our experimental model is IE1 protein, since it is highly expressed from day 1 to day 3 and then decreased at day 4 post-infection of HepG2 cells (Fig. 1D). In agreement with increased expression of IE1 protein, IE1 transcripts are detected as early as 2 hours post-infection and up to day 6 post-infection (Fig. 1E). In contrast, we did not detect significant levels of US28 protein and transcript following infection of HepG2 cells with HCMV (Figs. 1D and 1E). Although we cannot exclude a role of US28 protein in IL-6 production and STAT3 activation in PHH, IE1 protein is the most likely candidate to explain IL-6-STAT3 activation in HepG2 cells infected with HCMV.

Cyclin D1 is an important cell-cycle regulatory protein that is required for completion of the G1/S-phase transition in normal mammalian cells, and cyclin D1 gene expression is controlled by activated STAT3 [31], [42]. Overexpression of cyclin D1 mRNA and protein has been observed in several types of solid tumors, including HCC, and is associated with the early onset of cancer and aggressive tumor progression [42], [43]. Cyclin D1 is also intimately involved in resistance to apoptosis, making it an attractive therapeutic target for controlling tumor growth [44]. CADPE, a compound with known antioxidant properties, antagonizes IL-6, strongly suppressing STAT3 phosphorylation/activation and inhibiting cyclin D1 transcription in HCC cells [31]. Finally, blocking STAT3 activation with decoy-ODN, a specific inhibitor of activated STAT3, inhibits the growth of human HCC cells [38].

In addition to the cyclin D1 gene, STAT3 activates several genes involved in cell cycle progression, such as fos, myc, and pim-1, and up-regulates anti-apoptotic genes such as Bcl-2 and survivin [9], [10]. Survivin, a member of the inhibitor of apoptosis protein (IAP) family of proteins, is frequently expressed in human tumors, including HCC [32], [45]. Interestingly, IL-6 secreted by endothelial cells infected with HCMV promotes cell survival by stimulating survivin expression [46]. In agreement with these data, we observed that survivin was upregulated in HCMV-infected HepG2 cells and PHH in parallel with STAT3 activation. In agreement with our data, survivin is expressed in most HCC cases, and its expression in HCC correlates significantly with low-grade tumors, expression of cyclin D1, and phospho-STAT3, and is inversely associated with apoptosis [45].

Interestingly, despite the proliferation status induced by HCMV, we observed an apparently appropriate activation of the antitumor protein p53 and one of its main effectors, the protein p21waf, in HepG2 cells and PHH infected with HCMV. The tumor suppressor protein p53 responds to a wide variety of cellular stress by inducing cell cycle arrest or by triggering apoptosis. In unstressed cell, p53 expression is inhibited by the protein Mdm2, whereas p53-Mdm2 interaction is disrupted in stressed cells, leading to p53 activation [47]. P53 expression and/or functions are regularly altered in cancers [33]. Previous studies have noticed that HCMV induced an over-expression of p53 in several cell types *in vitro* [48]–[50]. This p53 over-expression was partly due to a down-regulation of the p53-inhibitor Mdm2 which began 24 hours post-infection, in accordance with our observation [51]. Nevertheless, p53 functions were altered in some HCMV-infected cell types. P53 was sequestrated in the cytoplasm of endothelial cells infected with HCMV, contributing to the HCMV-induced resistance to apoptosis [48]. Moreover, the immediate-early 2 protein (IE2) of HCMV down-regulates the transactivation function of p53 *in vivo* [52]. The p21 protein has been considered for a long time as one of the most important mediator of the antitumor effect of p53 by repressing cell cycle progression [53]. However, recent studies have highlighted a p21 accumulation (predominantly in cytoplasm) and a tumorigenic role of p21 in some cancers, that may rely to its ability to suppress apoptosis and to promote the assembly of cyclin D1 with cyclin-dependant kinases 4 and 6 [53]. Interestingly, p21 expression was enhanced in cancer cells from patients with HCC, especially in moderately and poorly differentiated cancers, and p21 overexpression was recognized as an independent factor for HCC development in cirrhotic patients [54], [55]. The overexpression of p21 induced by HCMV in HepG2 cells and PHH may contribute to the initiation or to the promotion of HCC.

We also report for the first time that HCMV infection of PHH favors the appearance of colonies in soft agar. This assay is an anchorage-independent growth assay that is considered the most stringent assay for detecting the malignant transformation of cells. Thus, our data indicate that the HCMV strains HCMV-DB and AD169 allow the transformation of PHH, indicating that HCMV could directly trigger the transformation process. We also observed that the HepG2 cell line, which is derived from the liver tissue of a fifteen-year-old male with differentiated HCC, formed colonies in soft agar. In addition, colony formation was increased further in HCMV-infected HepG2 cells, suggesting a potential role for HCMV as an oncomodulator [19], [20].

In recent years, multiple reports have shown that subpopulations of so-called cancer stem cells (CSCs) are required for sustained tumor growth and progression, and may be responsible for cancer recurrence and metastasis [56]. The IL-6-STAT3 axis has been reported to drive the conversion of non-stem cancer cells into CSCs in several human cancers [57], [58]. The expansion of CSCs can be measured by the formation of tumorspheres and STAT3 activation has been shown to be critical for neurosphere formation in glioblastoma and tumorsphere formation in human colon cancer cells [58], [59]. Recently, HepG2 cells have been shown to form tumorspheres in stem cell conditioned culture medium [35]. Our data indicate that HCMV infection of HepG2 cells enhances further the tumorsphere formation, and indicate that HCMV might act as an oncomodulator in already transformed HepG2 cells.

We previously reported that there was a higher incidence of HCMV DNA in biopsies from HCC patients than in biopsies from normal control patients [7]. These data further indicate that HCMV could play a significant role in the etiology of HCC, similar to its role in glioblatoma and medulloblastoma development [12], [17]. The anti-cancer kinase inhibitor sorafenib inhibits replication of HCMV at clinically relevant concentrations and, in contrast to ganciclovir, suppresses HCMV immediate-early antigen (IEA) expression, which is involved in IL-6 production [60]. Interestingly, expression of STAT3-driven genes, including cyclin D1 and survivin, is repressed by sorafenib in HCC cells [61]. Therefore, sorafenib or sorafenib derivatives could block the expression of HCMV IEA and thus block the IL-6-JAK-STAT3 axis that leads to cell proliferation and resistance to apoptosis in HCC. Interestingly, it was reported recently that sorafenib can induce complete histologic responses in advanced HCC [62].

In conclusion, our findings suggest that HCMV might play an unexpected and key role for the initiation and promotion of HCC, and raise the possibility that anti-HCMV-based strategies could improve the management of this disease.
