# Correction: HCMV Activates the IL-6-JAK-STAT3 Axis in HepG2 Cells and Primary Human Hepatocytes

**DOI:** 10.1371/annotation/35a1ab77-2ece-4fc7-9f1e-11c276f9d7c8

**Published:** 2013-05-29

**Authors:** Quentin Lepiller, Wasim Abbas, Amit Kumar, Manoj K. Tripathy, Georges Herbein

The authors would like to add the following clarifications regarding Figures 2 and 3 in the article:

- In Fig. 2C showing the expression of IE1 and US28 transcripts in HepG2 cells, the β-globin panel is shown in duplicate below the IE1 and US28 panels to facilitate the results reading.

- In Fig. 3A showing pSTAT3, STAT3 and β-actin expressions in PHH at Day1 (PHH section, middle panel), a band in the original blot for pSTAT3 and β-actin corresponding to another simultaneously tested (unmentioned) parameter was masked to prepare the final figure. An empty space in the original blot for STAT3 was masked to prepare the figure. Original non re-arranged blots are provided.

- In Fig. 3C showing pSTAT3 and β-actin expressions in HepG2 cells in the presence of JAK/STAT3 inhibitors (HepG2 section, left panel), the original blot was re-arranged to mask additional bands corresponding to other simultaneously tested (unmentioned) parameters. Original non re-arranged blot is provided.

Fig. 3A: http://www.plosone.org/corrections/pone.0059591.g003a.cn.tif

Fig. 3C: http://www.plosone.org/corrections/pone.0059591.g003c.cn.tif

